# Geroprotectors: A Unified Concept and Screening Approaches

**DOI:** 10.14336/AD.2016.1022

**Published:** 2017-05-02

**Authors:** Alexey Moskalev, Elizaveta Chernyagina, Anna Kudryavtseva, Mikhail Shaposhnikov

**Affiliations:** ^1^Laboratory of postgenomic studies, Engelhardt Institute of Molecular Biology of Russian Academy of Sciences, Moscow, 119991, Russia; ^2^Laboratory of genetics of aging and longevity, Moscow Institute of Physics and Technology, Dolgoprudny, 141700, Russia; ^3^Laboratory of molecular radiobiology and gerontology, Institute of Biology of Komi Science Center of Ural Branch of Russian Academy of Sciences, Syktyvkar, 167982, Russia

**Keywords:** aging, criteria of geroprotectors, geroprotectors, healthspan, lifespan

## Abstract

Although the geroprotectors discovery is a new biomedicine trend and more than 200 compounds can slow aging and increase the lifespan of the model organism, there are still no geroprotectors on the market. The reasons may be partly related to the lack of a unified concept of geroprotector, accepted by the scientific community. Such concept as a system of criteria for geroprotector identification and classification can form a basis for an analytical model of anti-aging drugs, help to consolidate the efforts of various research initiatives in this area and compare their results. Here, we review the existing classification and characteristics of geroprotectors based on their effect on the survival of a group of individuals or pharmaceutics classes, according to the proposed mechanism of their geroprotective action or theories of aging. After discussing advantages and disadvantages of these approaches, we offer a new concept based on the maintenance of homeostatic capacity because aging can be considered as exponential shrinkage of homeostatic capacity leading to the onset of age-related diseases and death. Besides, we review the most promising current screening approaches to finding new geroprotectors. Establishing the classification of existing geroprotectors based on physiology and current understanding of the nature of aging is essential for putting the existing knowledge into a single system. This system could be useful to formulate standards for finding and creating new geroprotectors. Standardization, in turn, would allow easier comparison and combination of experimental data obtained by different research groups.

Aging is a complex biological process affecting molecular, cellular, tissue, organ, system, organismal and even psychological organism’s levels [1, 2]. The cause and effect relationships and interactions between aging processes on all levels accumulated on the platform Aging Chart (http://agingchart.org/) [3]. Aging causes disease progress and a gradual decline in physical and mental function. Because of the rapid aging of the population, the risk of economic collapse in developed countries is increasing [4]. Therefore, anti-aging and disease prevention become a high priority science challenge.

Although the geroprotectors discovery is a popular biomedicine trend and more than 200 compounds can slow aging and increase the lifespan of animal models according to the Geroprotectors.org database [5], there are still no geroprotectors on the market. The reasons may be related to the lack of a unified concept of aging mechanisms, the problem of translation of geroprotectors studies results from model organisms to humans, low level of interest from big pharma since aging has no status as a disease [3, 6]. But one of the main obstacles, in our opinion, is the lack of a concept of geroprotector accepted by the scientific community. Such concept as a system of criteria for geroprotector identification and classification can form the basis for an analytical model of geroprotectors, help consolidate the efforts of various research initiatives in this area and compare their results. This model can serve as a platform for formulating and solving a variety of tasks, from a selection of the most promising and efficient existing candidate geroprotectors to possible constructing of a model geroprotector that can be searched in the libraries of compounds or synthesized purposefully.

## Definition of geroprotector and the system of its evaluation criteria

The founder of scientific “gerontology” is famous Russian and French biologist and Nobel laureate Ilya Mechnikov, who first used the term “geroprotector” [7]. The literal translation of "geroprotector" is "protecting against aging". From the initial determination, the primary criterion of geroprotector is the ability to increase the lifespan of model organisms.

A full monose mantic definition of geroprotector should be represented by a system that characterizes geroprotectors in a complete form. Such system could not limit to the requirement of lifespan increasing. For example, uncoupling lifespan and healthspan are possible, as in the case with *C. elegans* longevity mutants [8]. Therefore, requiring from geroprotector not only lifespan extension but also healthspan prolongation, the criterion of lifespan expectancy (LE) increase should be supplemented by criterion of maintaining a high quality of life and others. In previous paper [9] we proposed such system of evaluation criteria to define geroprotector candidates:

### Primary selection criteria for potential geroprotectors:

1. The most significant main rule for geroprotectors is evidently the ability to increase lifespan.
2. Candidate geroprotectors should ameliorate molecular, cellular, and physiological biomarkers to a younger state or slow the progression of age-related change in these markers.
3. The therapeutic lifespan extending dose of geroprotector should be several orders of magnitude less than the toxic dose.
4. Potential geroprotectors should improve health-related quality of life: physical, mental, emotional, and social functioning of the treated person.

### Secondary selection criteria for potential geroprotector:

5. The target or mechanism of action of the geroprotector should be evolutionarily conserved.
6. Reproducibility of geroprotective effects on different model organisms increases the possibility of effects will also be discovered in humans, even in the absence of a known conserved target.
7. Candidate geroprotectors should be able to delay the progress of one or several age-associated disorders.
8. Potential geroprotectors should increase the organism resistance to unfavorable environmental factors.

The compliance of a substance with at least the majority of these criteria allows the claim that we are dealing with a candidate geroprotector. With the help of modern mathematical tools for data analysis and decision-making, such a system would facilitate formulating and solving a number of important scientific and applied problems, the most significant of which include:

- Selection of geroprotectors with the largest and most reliable effect on life expectancy.
- *A priori* selection of geroprotectors for in-depth experimental studies.
- *A priori* selection of geroprotectors for human clinical trials.
- Selection of the optimal compound of geroprotectors with maximum effect in animals.
- *A priori* selection of a complex of geroprotectors for human clinical trials.
- Sorting of geroprotectors into specific categories and searching for the best substances in each class.
- Building a model of the ideal geroprotector, and searching for such material or its synthetic preparation.

## The existing classification and characteristics of geroprotectors

The variety of potential geroprotectors requires their systematization and classification. The current approaches classify geroprotectors by the influence on the lifespan of a population, by the mechanisms of action on the processes of aging, and by the origin of active ingredients.

Nikolay Emanuel and Lyudmila Obukhova [10] proposed a classification of geroprotectors based on their effect on the survival of a group of individuals. According to this classification, all means of increasing LE can be divided into three groups:

- a geroprotector that increases the LE of all members of the population, thus increasing both the average and the maximum lifespans;
- geroprotectors that diminish the rate of extinction of long-lived individuals, thus leading to a significant increase of maximum lifespan;
- geroprotectors that increase the LE of the short-lived subpopulation, thus increasing the average LE without changing the maximum lifespan.

The main advantage of this classification is that it is based on an objective indicator that allows for accurate measurement - life expectancy, as well as taking into account the differences in the geroprotector’s effect in separate subpopulations. However, this classification has obvious limitations. It is applicable only when the mortality data are available for the entire study population. Very lengthy and costly experiments are required to collect such data. When human populations are concerned, even if such a study can be conducted, its results will become available only after decades of gathering data. Furthermore, to obtain statistically reliable estimates of the changes in the survival curve shape (i.e. the distribution of lifespan in the population), the large sample sizes, not less than 200 individuals in each the control and experimental groups, are required. Therefore, the cost of such experiments is high even when they are performed on relatively short-lived species. This approach does not take into account the effects of the geroprotector based on the intra-vital measurements at the individual or population levels, such as the state of functional systems, performance level, reproductive capacity, the incidence of diseases. Finally, this classification cannot be considered complete since it does not include some theoretically possible changes in the survival curve. If influence of some factors increases LE both for the population on average and for the greater part of its initial strength, this factor can be considered geroprotective, even if in a smaller portion of the population a decrease in LE is observed. The idea of building of geroprotector classifications based on the differences in the forms of survival curves is futile as geroprotectors may have different effects in different age periods. Furthermore, the populations may include groups that react differently to the geroprotector regardless of age. Therefore, the variability of changes in the lifespan distribution across the population expressed as changes in the survival curve can take an infinite number of forms, some of which can be rather unusual. For example, the distribution of lifespan may become bi- or polymodal under the influence of a geroprotector. It 's hard to draw a conclusion about the functional reasons for such variations by the survival curves' appearance. Therefore, this classification attempt seems to be unproductive regarding explaining the mechanisms of geroprotectors action. Moreover, such classification would be very complicated regarding reliable differentiation of the survival curve variants. This task would require enormous sample sizes and the use of very sophisticated mathematical methods.

Classification based on the features of the geroprotectors effect in different age ranges raises the question about the reasons for such differences like survival curve shifts for various geroprotectors. With the same populational effects, the causes may be quite different. These reasons can be both endogenous (differences in targets and mechanisms of geroprotectors action) and exogenous (due to the difference in external factors). The proposed classification does not provide answers to these questions.

Vladimir Anisimov [11] suggested dividing geroprotectors into two groups according to the proposed mechanism of their geroprotective action:

- I group - drugs that prevent accidental damage to macromolecules;
- II group - drugs or factors slowing the implementation of the genetic program of aging and the formation of age-related pathologies.

He also highlighted several types of geroprotectors using the most recognized theories of aging at that time as the typing criteria [11]:

- antioxidants;
- inhibitors of cross-linking;
- neurotropic substances;
- hormones (growth hormone, thyroid hormones, adrenocortical hormones, sex hormones and contraceptives, melatonin and peptides of pineal gland);
- antidiabetic agents;
- immunomodulators;
- mimetics of caloric restriction;
- entero- sorbents;
- adaptogens;
- other substances and factors.

This approach takes into account the significant differences in the modifying influence of geroprotectors on physiological functions of the body and reproductive system. It also considers the mechanism of the drug's actions, making it possible to personalize the approaches to the prevention of aging by diagnostic data of the patient. However, it does not take into consideration the effects of the regulatory network of processes associated with aging. Anisimov’s classification is too multi-leveled, with the boundaries between the geroprotector types often blurred. For instance, antioxidants may be hormones (melatonin [12]) adaptogens (polyphenols [13,14]) or immunomodulators (lipoic acid [15]). Geroprotectors that don’t fit the selected types are classified as "other substances". The principle of classification consistency implies that all classes of substances represent disjointed sets. However, some data contradict this classification. For example, high levels of some hormones the production of which declines with age (such as growth hormone and insulin-like peptides) are associated with accelerated aging in most model organisms [16]. Alpha-tocopherol, a powerful membrane antioxidant did not show any geroprotective influence [17]. On the contrary, some strong pro-oxidants, such as paraquat, are known to have geroprotective effects in model animals [18]. This discrepancy is consistent with the new concept of mitohormesis, according to which the increase of the level of mitochondrial free radicals to a certain extent activates the cells' resistance to stress and thus slows down the aging [19]. Thus, the above classification of geroprotectors needs to be updated.

## Further attempts to classify geroprotectors

Vijay Kapoor and coworkers [20] divided geroprotectors into three large groups: natural products with reported anti-aging effects, synthetic drugs with anti-aging activity and hormone replacement therapy. Apparently, this approach is based on the sources of the substances with geroprotective effect and does not take into account mechanisms of their action on the body and the aging process.

In one of the latest attempts, Maria Carretero and coworkers [21] classified the compounds extending the lifespan of *C. elegans* into pharmaceutics classes: antioxidants, metabolites, kinase inhibitors, regulators of nuclear receptors of hormones, G-protein coupled receptor ligands and natural substances. This classification was introduced to describe the ability of materials to extend the lifespan in *C. elegans* by their known pharmacological mode of action in humans. However, this example of classification does not take into consideration the known mechanisms of aging and longevity.

Establishing the classification of existing geroprotectors based on physiology and current understanding of the nature of aging is essential for putting the existing knowledge into a single system. This system could be useful to formulate the standards for finding and creating new geroprotectors. Standardization, in turn, would allow easier comparison and combination of experimental data obtained by different research groups.

## Classification of geroprotectors based on the concept of homeostasis

Aging can be considered as exponential shrinkage of homeostatic capacity leading to the onset of age-related diseases and death. Aging-associated loss of homeostasis is observed at different biological levels, and appears as a common denominator for different hallmarks of aging process [22, 23]. Thus, the primary anti-aging strategy could be based on the maintenance of homeostatic capabilities.

However, to date there are a little approach to quantitative evaluation of age-related changes of homeostasis and deficiency of experimental data regarding relationship between homeostasis parameters and life expectancy. Given that cellular and organismal homeostasis in the face of external perturbations and age-related changes is maintained by coordinated action of stress response and repair pathways [24, 25], the description of age-related changes in the activity of signaling pathways can be one of the indicators of violation of homeostasis. Recently Alex Zhavoronkov and coworkers proposed the method that evaluates the changes in the collection of activated or suppressed signaling pathways involved in aging and longevity using the gene expression data and epigenetic profiles of young and old patients' tissues [26]. This method can be used to assess the homeostatic capacity in the aging organism. However further experimental studies are needed to study the relationship between homeostatic capacity and life expectancy.

By this idea, we have classified the potential geroprotectors according to their ability to maintain homeostasis, thereby providing for healthy longevity:

### Suppression of the consequences of homeostasis disruption

With age, the disturbances of homeostasis manifest themselves in the deviations of such vital parameters as acid-base balance in blood, blood pressure, blood glucose and cholesterol levels from the healthy norm. Therefore, the substances that prevent the development of such aging-associated conditions can be seen as geroprotectors. Antidiabetic, anti-arrhythmic, lipid-lowering, cardiovascular and antihypertensive drugs can be considered geroprotectors as such. For example, metformin, an oral antidiabetic agent of the biguanide class, can prolong the life of *C. elegans* [27], *D. melanogaster* [28] and *M. musculus* [29]. Bezafibrate, the lipid-lowering drugs used to control the level of cholesterol and triglycerides in the blood, has a geroprotective effect on *C. elegans* [30].

At the cellular level, homeostatic disturbances reveal themselves in a process called cellular senescence. Recently a new class of drugs, senolytics, was chronicled to have selectively killed senescent cells. In eliminating senescent cells dasatinib and quercetin showed notable potential [30].

### Enhancement of homeostatic systems

At the cellular level a key homeostatic role is played by proteins of the stress response. Lack of nutrients, DNA damage, and disturbances in the proteostasis are perceived as healthy stressors by the cell. The activation of stress resistance system cannot only reverse the damage, but also transfer the system to a higher level of protection against new spontaneous errors and damage [2]. Stress resistance mechanisms can be induced by agents causing moderate stress which is not accompanied by a significant injury but is capable of activating the protective response. This phenomenon was termed "hormesis" [31], and its causative agents received the name “hormetins” [32]. Quercetin [33], a widespread natural flavonol that increases the lifespan and stress resistance in experiments on *C. elegans* [34], is an example of a hormetin. Curcumin which extends the lifespan of *Drosophila* also appears to act as hormetin, as it weakly denatures proteins and thus increases the stress resistance [35].

Age-related dysregulation of gene expression is one of the fundamental causes of homeostasis disruptions. These changes occur as a result of epigenetics drift [36], and its prevention and the return of the transcriptome parameters back to the norm is a potential mechanism of geroprotection [26, 37]. The Mimetics of caloric restriction (the compounds that deplete acetyl coenzyme A, inhibit acetyltransferases or stimulate the activity of deacetylases) influence the epigenetic state of the cells, return the expression of genes associated with aging back to normal levels, and increase the resistance to stress [38]. Nicotinamide riboside [39, 40] is an example of geroprotectors from the class caloric restriction mimetics. This compound is a biochemical precursor of vitamin B3 and can increase the replicative lifespan of *S. cerevisiae* [41]. Resveratrol, a nonspecific activator of deacetylase Sir2 in yeast, demonstrates the geroprotective effect in this model organism [42]. Epigenetic drugs such as trichostatin A and 4-phenylbutyrate, the inhibitors of histone deacetylase HDAC, increase the lifespan of fruit flies [43, 44].

### Neutralization of damaging agents that causes disruption of homeostasis

The damage to macromolecules, such as oxidative carbonylation [45] and non-enzymatic glycosylation [46] of proteins, is considered a primary cause of aging processes. This damage may lead to the formation of protein aggregates such as amyloids that are difficult to remove. Oxidative carbonylation is caused by highly reactive hydroxyl radicals OH^•^ formed in the presence of Fe^2+^ via the Fenton reaction [45]. Transition metal ions (Cu^2+^, Fe^2+^, and Zn^2+^) are involved in catalysis of sugar autoxidation, glycoxidation, cross-linking and can stimulate the formation of certain advanced glycation end-products (AGEs) in the Maillard reaction [47-49]. For example, the two most commonly measured AGEs, N^ε^-(carboxymethyl) lysine and pentosidine, are formed by sequential glycation and oxidation reactions wich may be catalysed by the transition metal ions [49, 50].

This group of geroprotectors includes:

- chelators of Cu and Fe, for example, EDTA which can extend the lifespan of *C. elegans* [51], and *R. norvegicus* [52];
- ROS-scavengers, as exemplified by ethoxiquin which extends the lifespan of *M. musculus* [53];
- compounds that attenuate the formation of advanced glycation end products, for example, a polyphenol plant butein that is known to increase the replicative lifespan of *S. cerevisiae* [42], and 1,2,4-triazolo [1,5-a] pyridines that prolong the lifespan of nematodes [54];
- anti-amyloid agents, such as polyphenols and curcumin which seem to inhibit the formation of amyloids from different proteins [55-59]. Tetrahydrocurcumin and green tea polyphenols extended the lifespan of C57BL/6 mice [60] but curcumin and tea polyphenols failed to extend the lifespan of F1 hybrid mice [61].

### Suppression of excessive homeostatic reactions that lead to even greater loss of homeostasis

Hyperfunction of some homeostatic reactions in response to stress may cause an even larger damaging effect than the original injury. For example, excessive activation of the enzyme PARP1 which recognizes DNA damage may lead to the depletion of cells' energy sources, chronic inflammation, and reduced LE [62]. Hyperactivation of the inflammatory process required to activate the immune function contributes to the acceleration of aging processes [63].

According to the geroconversion hypothesis of Mikhail Blagosklonny, senescent cells continue futile growth after the cell cycle arrest. Geroconversion leads to the hypersecretory, hypertrophic and pro-inflammatory cellular phenotypes that depend on mTOR kinase activity [64].

Thus, inhibition of overreaction may underlie geroprotection. Anti-inflammatory drugs such as ibuprofen, a propionic acid derivative, and a non-steroidal anti-inflammatory agent used to relieve pain, fever, and inflammation, fit well into this class of geroprotectors. This drug extends lifespan in experiments on *S. cerevisiae, C. elegans* and *D. melanogaster* [65]. Aspirin has proven to extend the lifespan of male mice. In addition, there’s good epidemiological evidence that aspirin delays the progression of several age-related diseases such as atherosclerosis, several cancers, and protective effects against neurodegenerative diseases such as Alzheimer’s and Parkinson’s disease [66].

The drugs aimed at specific targets hyperactivated during aging, such as mTORC1, NF-κB, PARP1, iNOX, COX2, and p38, can also be considered as inhibitors of hyperfunction. For example, rapamycin and its derivative everolimus, which acts as an inhibitor of mammalian target of rapamycin (mTOR), can extend the lifespan of *D. melanogaster* [67] and mice [68].

Our approach to the classification of geroprotectors has both advantages and disadvantages. This method is based on the mechanisms of action of geroprotectors and the mechanisms of aging itself and thus allows for a personalized approach to the prevention of accelerated aging. Such personalized approach would take into account the fundamental causes of aging prevailing in a particular patient. At the same time, certain geroprotectors (e.g., rapamycin, metformin) may still be allocated into several groups in this classification. However, this is most likely caused by the multi-functionality of targets or the existence of multiple objectives for the substances in question.

## Classification of approaches to finding new geroprotectors

A variety of approaches to finding new geroprotectors can be envisaged. Below we have listed the methods we consider the most promising.

### Screening approach

The object of testing is libraries of natural and synthetic compounds approved by the FDA [42, 69, 70]. For example, in the work of Linda Buck and Michael Petrascheck the libraries of compounds with known pharmacological properties towards mammals were screened to identify the substances capable of extending the lifespan of nematode *C. elegans*. Of the 1280 tested compounds, 60 compounds were found to increase the lifespan of nematodes. Among them, 33 compounds also increased the resistance to oxidative stress. Many compounds from the screened libraries are approved by the FDA for use in humans.

The advantage of the screening approach is the absence of the need to know the mechanisms of aging and potential targets. The disadvantages include the consumption of significant resources and low success rates.

To identify drugs that could postpone aging but do not have undesirable side effects, the "reverse pharmacology" approach can be used. In this method, the compounds are first screened for the ability to target various aging-related proteins, and then the identified substances are tested for the capacity to influence aging.

In addition to this approach, the "direct pharmacology" method can be employed. In this case, the compounds are tested directly *in vivo* for the presence of ability to delay aging or manifestations of age-related phenotypes. However, aging and LE reflects the phenotypes of the whole organism, which makes the *in vivo* screening in mammals a lengthy and expensive process [71].

### Homeostatic approach

This method relies on the search for physiological and biochemical abnormalities in the levels of hormones and the micro- and macronutrients in the body observed during the aging. Monitoring and maintaining an optimal level of hormones, cytokines, growth factors and macro-and micronutrients could become a practical application of this approach. For example, it was found that morphogen BMP-11/GDF11 is needed for normal functioning of the heart and skeletal muscles. The blood levels of BMP-11/GDF11 decrease with age, but their replenishment slows down the aging [72, 73]. Although it should be mentioned that a recent study failed to find lifespan extension from GDF11 administration in an accelerated aging mouse model. The results that GDF11 administration may reverse cardiac hypertrophy have also been questioned [74, 75].

The advantage of the homeostatic approach is that its results are the most relevant to a person as an object of study. The disadvantages include the systemic effects of hormones and nutrients with the possibility of unpredictable side effects.

### Mechanism-based approach

There are several different theories of aging, each of which focuses on an aspect associated with aging. Historically an important role was played by the Mechnikov’s theory of self-poisoning [7], the Harman's free radical theory [76], the theory of the stimulating effect of moderate stresses (hormesis) [77], telomeric hypothesis of aging [78, 79], and the theory of inflammation [80]. Geroprotective properties of entero- sorbents [81], antioxidants [82], hormetins [83], telomerase activators (TA-65) [84], and anti-inflammatory drugs [65, 85] are studied in connection with these concepts.

### Targeting approach

As mentioned above, 1825 genes whose knockout, knockdown or overexpression lead to an increase in LE in various model organisms are known at present [86]. The inhibitors or activators of these target genes and their encoded proteins might potentially be geroprotectors [87]. For each particular target gene or protein, a small molecule [88, 89], antibody [90], aptamer [91], microRNA [92] or shRNA [93] may be selected.

### The search for gene signatures

The approach consists of searching for specific patterns of expression for known geroprotective influences in the transcriptome and identification of such profiles of genes under the effect of different compounds. The comparison of transcriptome signatures between animals subjected to either caloric restriction or potential calorie restriction mimetics is an example of such an approach [94].

### Search for aging-related pathways

This is an approach aimed at multiple targets associated with established aging-related molecular pathways. The method consists of analysis of the transcriptomes, proteomes or metabolomes after potential geroprotective exposure. Such analysis would attempt to identify the suppression or activation patterns in the range of molecular pathways associated with triggering the aging mechanisms and the mechanisms of increasing the LE [26, 95].

### Analysis of cluster distances

In this approach the cluster distances between the reference and experimental transcriptomes/proteomes/metabolomes influenced by the potential geroprotector are calculated [96]. Relevant cell profiles of young people, super-centenarians, as well as extremely long-lived, cancer-free or extremely stress-resistant organisms might be used as reference data.

The clusters closest to the reference values are considered to potential geroprotectors. The main advantage of the approach is the absence of the need to find distinct molecular pathways or aging-associated genes. The method provides the possibility to view and analyze a complete set of changes.

## A single analytical model of geroprotector based on these criteria and classification

Development of a model of geroprotector involves the building of appropriate databases, which gather genomic, transcriptomic, metabolomic and proteomic data. Such bioinformatical model based on different approaches and methods of searching for gene signatures, identifying the aging-associated pathways, analyzing of cluster and machine learning will allow efficient conduction of the predictive part of the search for geroprotectors *in silico*.

The appropriate system of criteria and classification can help to develop geroprotectors’ databases, efficiency ratings and advise the approaches to predict and to model the geroprotective properties. The applied value of the finding, testing and classification of geroprotectors is their considerable potential for prevention and treatment of age-related pathologies through acting on the fundamental cause of these diseases - the process of aging. Ultimately, they can help in achieving longevity and radical extension of the fully active period of human life.
